# Persistent sciatic artery an incidental finding

**DOI:** 10.1016/j.radcr.2021.03.057

**Published:** 2021-05-03

**Authors:** Adil Omer, Maitham Alkadumi, Sandhya Jupalli, Joseph Dobtsis

**Affiliations:** Department of Radiology, NYC Health + Hospitals | Harlem, New York, USA

**Keywords:** Sciatic artery, Aneurysm, Persistent

## Abstract

Persistent sciatic artery is a direct continuation of the internal iliac artery. It is a rare anatomic variant where the sciatic artery does not regress during fetal development. We present a case of a 43-year-old male with a right leg ulcer who has an incidental finding of persistent sciatic artery on Computed Tomography (CT) of the thigh with Intravenous (IV) contrast to evaluate for abscess formation. A major complication seen in some patients with persistent sciatic artery is aneurysm formation which predispose patients to limb-threatening thromboembolism. Asymptomatic cases such as this case require no intervention apart from follow up.

## Introduction

Sciatic arteries are the main blood supply to the lower limbs during fetal development. The sciatic artery eventually regresses leaving remnants that persist as popliteal and peroneal arteries. Before sciatic regression, the popliteal and peroneal arteries establish continuity with the superficial femoral artery [1,2]. Persistent sciatic artery is a direct continuation of the internal iliac artery. It is a rare anatomic variant where the sciatic artery does not regress during fetal development [1].

## Case report

**Fig. 1:**
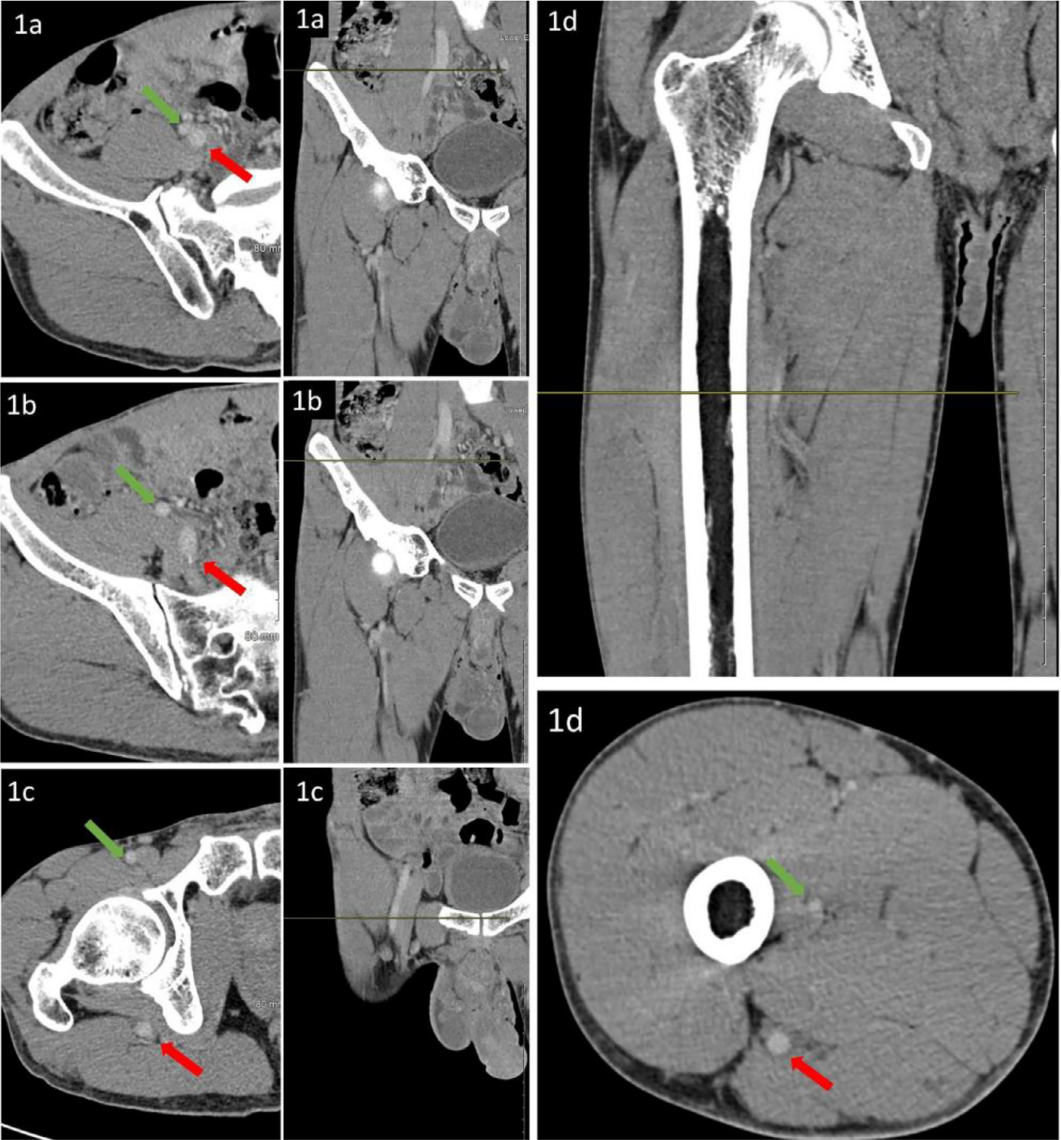
A 43-year-old male presented with a right leg ulcer. (1A) through (1D) represent consecutive cross-sectional images with corresponding levels marked on the sagittal plane from the origin of the persistent sciatic artery to mid-thigh in the cranio-caudal direction. The green arrow follows the External Iliac artery, common femoral artery, and the superficial femoral artery. The red arrow follows the internal iliac artery as it continues as the persistent sciatic artery.

**Fig. 2:**
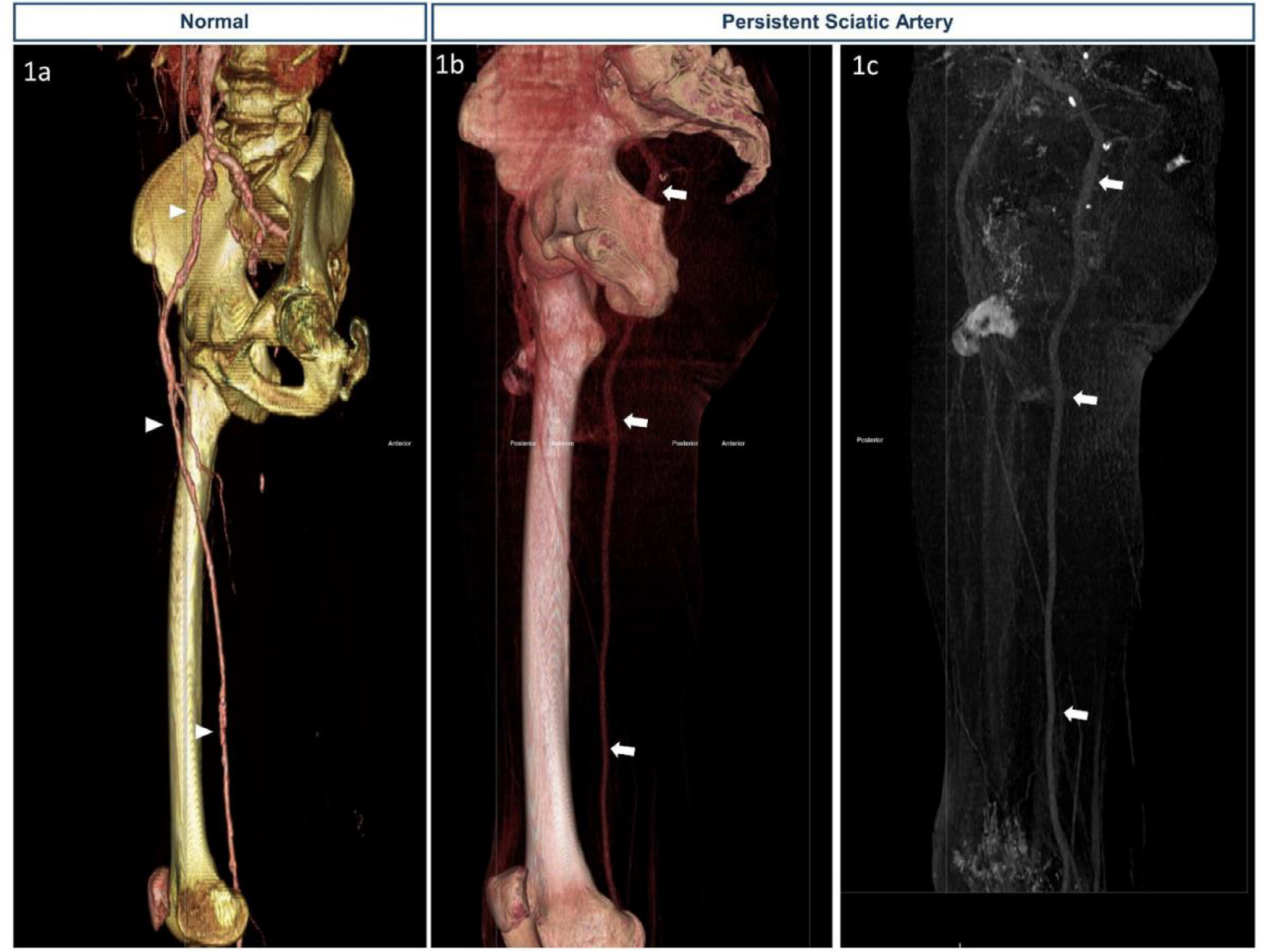
A 43-year-old male presented with a right leg ulcer. Findings: (1A) is a CTA 3D reconstruction of the right lower extremity demonstrating the normal anatomy of the common femoral artery, superficial femoral artery, and its distal continuation as the popliteal artery provided for comparison (arrow head). (1B) and (1C) 3D reconstruction of CT of the right femur with contrast demonstrating persistent sciatic artery with hypoplastic superficial femoral artery.

A 43-year-old male without significant past medical history, including absence of history of peripheral vascular disease presents with a right thigh ulcer. CT scan of the right thigh with contrast is performed to evaluate soft tissue involvement. Incidentally found is a persistent sciatic artery (PSA), type 2a (Fig. 1 and Fig. 2). PSA type 2A is arterial continuation of the internal iliac artery coursing caudally through the major sciatic foramen. It continues deep to the gluteus maximus muscle mainly adopting a pathway within the posterior thigh compartment which follows the course of the sciatic nerve proximally. Distally, the artery continues as popliteal artery and branches as the anterior tibial, posterior tibial and peroneal arteries. The superficial femoral artery is patent but diminutive. (Fig. 1 and Fig.2). The sciatic nerve is visualized coursing along the sciatic artery best seen at the superolateral aspect of the sciatic artery (Fig. 1D). Contralateral limb involvement was not established due to unilateral imaging. This is an incidental finding in this patient without related symptoms, therefore no treatment is required. Follow up imaging may be considered.

## Discussion

Persistent sciatic artery is a rare anatomic variant where the sciatic artery persists through adulthood which predisposes patients to complications such as aneurysms and distal limb threatening thromboembolism [1,2].

There are 5 types of PSA. In type 1 and type 2, the PSA is completely present. The distinction between types 1 and 2 is that in type 1 there is a fully developed superficial femoral artery (SFA) and in type 2 the SFA is partially (Type 2a) or completely absent (Type 2b). The presented case is an example of type 2 PSA; SFA is partially developed which is subclassified as Type 2a. In type 3 and 4 categories, the superficial femoral artery is fully developed [3, 4]. Type 3 represents a partially proximally developed PSA, which is absent distally, and type 4 represents a partially distally developed PSA with a proximal absence of the artery [3]. Gaufre et al. [5] described type 5 PSA in which the PSA originates from the median sacral artery with complete SFA in type 5a and absent SFA in type 5b [Table 1]. Massignan et al. [6] reported 4 cases, 3 of which matched the presented case classification of type 2a pattern while case # 2 represented type 2b. Meo RD et al. [7] reported a case with bilateral type 2a PSA which is significant for symmetrical hypertrophy unlike the presented case which demonstrated relatively normal PSA caliber.

**Table 1:** Types of persistent sciatic artery.

	PSA origin	PSA	SFA
Type 1	Internal iliac artery	Fully developed	Fully developed
Type 2	internal iliac artery	Fully developed	Partially developed in Type 2a and absent in Type 2b
Type 3	internal iliac artery	Partially developed; proximally	Fully developed
Type 4	internal iliac artery	Partially developed; distally	Fully developed
Type 5	median sacral artery	fully developed	fully developed in type 5a and type 5b undeveloped

Persistent sciatic artery incidence is approximately 0.025%-0.04% [2]. Van Hooft et al. [3] reported Persistent Sciatic Artery (PSA) mean age of diagnosis is 57 years with an almost equal gender distribution; 56% were women and 44% were men based on his review of the literature [3]. Unilateral PSA represents 70% of PSA cases [4]. Aneurysm formation in PSA is estimated at 44% of cases [8].

The presentation is silent and usually diagnosed incidentally in most cases. Symptoms such as claudication occur with reduced blood flow due to hypoplasia of the SFA and insufficient sciatic artery. On physical examination absence of the femoral pulse in presence of the popliteal and pedal pulses likely to be the presentation in type 2 PSA due to SFA severe hypoplasia in type 2a or SFA absence in type 2b, however, commonly the SFA is patent and palpable [8]. Additionally, complications such as aneurysms may manifest as a pulsatile mass within the buttocks, Alsaadoun AR et al. *[9]* reported bilateral PSA with complicated symptomatic left sided PSA aneurysm. The left sided PSA resembled type 1 classification while the right resembled type 3 classification [9]. Moreover, aneurysms may compress the sciatic nerve giving rise to sciatica which presents with pain, numbness, or motor impairment [8].

Differential diagnosis includes gluteal artery aneurysm, an entity that simulates complicated aneurysmal PSA. Imaging modalities used to evaluate aneurysms include ultrasonography, computed tomography, magnetic resonance imaging, or angiography. Angiography is used for preoperative evaluation to map distal extremity circulation for possible reconstruction. Additional differential considerations for throbbing PSA aneurysm include enlarged bursae, abscess, sciatic hernia, granulomatous disease, neoplasms, and congenital or acquired arteriovenous fistula [8].

Asymptomatic incidentally diagnosed PSA doesn't require intervention. However, serial physical examination and noninvasive imaging of the PSA is an appropriate approach [8]. Aneurysms are treated by percutaneous transcatheter embolization or ligation (if sufficient collaterals or normal SFA) to avoid future complications such as limb-threatening thromboembolism or rupture [4, 8].

The significance of recognizing persistent sciatic artery lies in its tendency to develop aneurysms and subsequent limb-threatening thromboembolism. Additionally, knowledge of this entity changes the surgical approach during bypass grafting [4] (Table 2).

**Table 2:** Summary table.

Etiology	Embryological defect causing failure of sciatic artery regression [4]
Incidence	0.025%-0.04% [2]
Gender ratio	0.56: 0.44 female to male [3]
Age predilection	57 [3]
Risk factors	None identified
Treatment	Follow up for asymptomatic cases. Aneurysmal percutaneous stenting or ligation [8]
Prognosis	44% of PSA cases develop aneurysm [8]
Findings on imaging	persistent posterior sciatic artery [2-5,8]

## Human and animal rights

No experiments conducted.

## Patient consent

Did the author obtain written informed consent from the patient for submission of this manuscript for publication? (no.).The case was anonymized.
